# Mass Spectrometry-Based Proteomics for Classification and Treatment Optimisation of Triple Negative Breast Cancer

**DOI:** 10.3390/jpm14090944

**Published:** 2024-09-05

**Authors:** Essraa Metwali, Stephen Pennington

**Affiliations:** 1 School of Medicine, UCD Conway Institute for Biomolecular Research, University College Dublin, D04 C1P1 Dublin, Ireland; stephen.pennington@ucd.ie; 2 King Abdullah International Medical Research Center (KAIMRC), Ministry of National Guard, Jeddah-Makka Expressway, Jeddah 22384, Saudi Arabia

**Keywords:** triple-negative breast cancer (TNBC), neoadjuvant chemotherapy (NAC), pathological complete response (pCR), mass spectrometry (MS), multiple reaction monitoring (MRM)

## Abstract

Triple-negative breast cancer (TNBC) presents a significant medical challenge due to its highly invasive nature, high rate of metastasis, and lack of drug-targetable receptors, which together lead to poor prognosis and limited treatment options. The traditional treatment guidelines for early TNBC are based on a multimodal approach integrating chemotherapy, surgery, and radiation and are associated with low overall survival and high relapse rates. Therefore, the approach to treating early TNBC has shifted towards neoadjuvant treatment (NAC), given to the patient before surgery and which aims to reduce tumour size, reduce the risk of recurrence, and improve the pathological complete response (pCR) rate. However, recent studies have shown that NAC is associated with only 30% of patients achieving pCR. Thus, novel predictive biomarkers are essential if treatment decisions are to be optimised and chemotherapy toxicities minimised. Given the heterogeneity of TNBC, mass spectrometry-based proteomics technologies offer valuable tools for the discovery of targetable biomarkers for prognosis and prediction of toxicity. These biomarkers can serve as critical targets for therapeutic intervention. This review aims to provide a comprehensive overview of TNBC diagnosis and treatment, highlighting the need for a new approach. Specifically, it highlights how mass spectrometry-based can address key unmet clinical needs by identifying novel protein biomarkers to distinguish and early prognostication between TNBC patient groups who are being treated with NAC. By integrating proteomic insights, we anticipate enhanced treatment personalisation, improved clinical outcomes, and ultimately, increased survival rates for TNBC patients.

## 1. Introduction

Triple-negative is an aggressive subtype of breast cancer (BC) characterised by the absence of oestrogen receptor (ER), progesterone receptor (PR), and HER2 expression [1]. TNBC represents approximately 10% to 20% of all BC cases [2]. These tumours have a higher histological grade, an increased risk of locoregional recurrence, and greater metastatic potential [3]. Patients with TNBC also exhibit contralateral disease and systemic relapse at an earlier age, leading to poorer clinical outcomes and lower survival rates [3]. It is associated with a poor prognosis in terms of distant disease-free survival (DDFS) and overall survival (OS) compared to other subtypes of BC due to its aggressive nature and lack of recognised molecular targets for therapy [4].

There is a critical need to understand the molecular landscape of TNBC in order to identify biological characteristics that can predict response to current cytotoxic therapy and facilitate the development of targeted treatments. As specific treatment guidelines for TNBC are lacking, chemotherapy remains the primary treatment option for early and late-stage TNBC. The variable response of TNBC to conventional chemotherapy and the lack of effective targeted treatments have led researchers to focus on this subtype of BC [5].

Advancements in sequencing technologies have enabled comprehensive characterisation of the molecular features of tumours. TNBC is now recognised as a highly heterogeneous disease with diverse genomic and transcriptomic profiles. The significant heterogeneity of TNBC has made it challenging to identify suitable molecular targets in preclinical studies, and this challenge is reflected in the modest benefits observed from targeted therapies in clinical trials involving unselected TNBC patients. Consequently, efforts have been made to subtype TNBC based on biologically and clinically relevant characteristics. Subtyping TNBC based on actionable molecular features holds promise for identifying targeted therapies, improving clinical trial design, and stratifying patients [6].

Mass spectrometry (MS) has undergone significant advances in recent years, particularly in its application to cancer research. These developments have led to MS being routinely used in proteomics and to investigate a large variety of biological molecules [7]. Leveraging MS technology holds promise for advancing our understanding of TNBC biology, identifying actionable molecular features for targeted therapies, and stratifying patients for personalised treatment approaches to improve patient outcomes [8,9]. This review explores the role of MS-based proteomics in shedding light on the molecular landscape of TNBC and its implications for precision medicine in BC treatment.

## 2. Current Clinical Approach of Triple-Negative Breast Cancer

### 2.1. Breast Cancer Classification

There are numerous molecular variations contributing to BC carcinogenesis, leading to various classification systems. TNBC, the focus of this review, lacks the expression of ER, PR, and HER2 receptors. According to Perou et al., 2000 [10] described a 50-gene expression signature, identifying four distinct subgroups of BC: luminal A and luminal B, basal-like, and human epidermal growth factor receptor 2 (EGFR) (HER2) [2]. This classification shifted clinical management from focussing on tumour burden to a biology-centred approach. In addition, there is a growing consensus that optimal patient outcomes and an enhanced quality of life are achieved through the dedicated efforts of specialised multidisciplinary teams that rigorously adhere to well-defined treatment decision guidelines [2,11]. These guidelines, which play a pivotal role in shaping patient care, can be found within the framework of national clinical directives specifically designed to address the intricacies of BC diagnosis, staging, and treatment [12]. They draw upon the latest advances in medical research and knowledge, allowing medical professionals to deliver care that is both current and effective. Approximately 10% of BCs have an inherited component and are associated with a family history [13]. Driver mutations refer to DNA damage or sequence changes that confer a proliferative advantage to cells, allowing the growth of a neoplastic clone [14]. Women with mutations in the high penetrance tumour suppressor genes BRCA1 and BRCA2 have a 72% risk of developing BC by the age of 80. Furthermore, in a series of early-stage BC cases, several genes were found to be frequently mutated in tumour cells, including TP53 (41% of tumours), PIK3CA (30%), MYC (20%), PTEN (16%), CCND1 (16%), ERBB2 (13%), FGFR1 (11%), and GATA3 (10%) [2,11]. These genes encode cell cycle modulators that can be repressed, activated, or inhibited. Luminal A tumours showed a high prevalence of PIK3CA mutations (49%), while basal-like tumours (84%) were notable for TP53 mutations. However, as noted, there are multiple molecular drivers for TNBC within its subtypes, including TP53, PIK3CA, PTEN, and KMT2C [1]. Table 1 shows a summary of important genes commonly altered/overexpressed in TNBC.

### 2.2. Subclassification of TNBC: Molecular Insights

**Figure 1:**
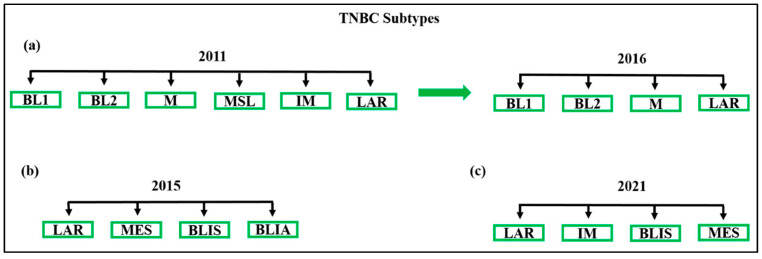
Comparison of molecular classifications of TNBCs and histological correlation. TNBC subtypes were identified using gene expression profiles. (**a**) In 2011, Lehmann et al. classified TNBC patients into six TNBC types: BL1, BL2, M, MSL, IM, and LAR. TNBC molecular subtypes were reclassified by Lehmann et al. in 2016 from six (TNBC type) to four (TNBC type-4) tumour-specific subtypes: BL1, BL2, M, and LAR [15]. (**b**) According to Burstein et al., four subtypes exist: LAR, MES, BLIS, and BLIA. (**c**) FUTURE trial design: LAR, IM, MES, and basal-like immune-suppressed trials (BLIS). Figure adopted from Hossain et al., 2021 [18].

Several attempts have been made to establish a molecular classification for TNBC. Initially, Lehmann et al. identified six TNBC transcriptional subtypes, each with distinct molecular characteristics and oncogenic signalling pathways [15]. These subtypes include immunomodulatory (IM), mesenchymal (M), mesenchymal stem-like (MSL), luminal androgen receptor (LAR), and two basal-like subtypes (BL1 and BL2) [16]. Figure 1 provides an overview of various TNBC subtype classifications. The BL2 subtype is characterised by enriched growth factor signalling and myoepithelial markers, while the BL1 subtype is characterised by elevated expression of cell cycle and DNA damage response genes [15]. The IM subtype is defined by the expression of immune antigen and cytokine genes, as well as immune signal transduction pathways, which likely represent gene expression from both tumour cells and infiltrating lymphocytes. The MSL subtype shows decreased expression of genes involved in proliferation, while both M and MSL subtypes exhibit increased expression of genes associated with epithelial-mesenchymal transition and growth factor pathways. The LAR subtype is defined by luminal gene expression and the expression of AR [17]. Over time, the original six subtypes have been consolidated into four (BL1, BL2, M, and LAR) based on the complexity and overlapping histological landscapes of tumour samples [18]. It has also been shown that the IM and MSL subtypes represent tumours with significant infiltration of lymphocytes and tumour-associated mesenchymal cells, respectively [17]. These TNBC subtypes have demonstrated significantly different responses to chemotherapy, suggesting the potential benefit of this classification in future clinical trial designs [18]. BL1 tumours demonstrate the highest response rates (52%), while BL2, LAR, and M tumours exhibit the lowest response rates (0%, 10%, and 23%, respectively) [1]. The identification of TNBC subtypes through genomic profiling has opened avenues for future clinical trials involving novel and targeted therapies [15]. However, due to the complexity and cost of whole genome analyses, these subtypes are not currently utilised routinely in cancer clinics [19]. Further research is necessary to validate and standardise therapies based on molecular subtypes. Modern TNBC treatment involves a multimodal approach that integrates systemic treatment, surgery, and radiation. It is crucial to consider oncological medication safety guidelines, which can be found in the National Cancer Control Programme [20], while tailoring the treatment strategy to the individual patient’s needs and preferences and striving to minimise treatment burden whenever possible [21].

As research advances in understanding TNBC’s molecular landscape, several key molecular targets have emerged that offer potential therapeutic avenues. These targets are closely linked to the distinct molecular subtypes of TNBC, as each subtype exhibits specific signalling pathways and biological processes. Table 2 shows a summary of current molecular targets in TNBC.

### 2.3. Diagnosis, Staging, and Current Treatment of TNBC

Locoregional therapy for early BC involves the surgical removal of affected axillary lymph nodes and the tumour, regardless of the molecular subtype [11]. TNBC is commonly associated with higher pathologic grades, is more prevalent in young African American women [28], and shows a strong association with BRCA pathogenic mutations. Despite advancements in systemic therapies, 20–40% of patients with early-stage TNBC develop metastatic disease within 3–5 years of diagnosis [29]. Unfortunately, the median OS for patients with metastases remains approximately 20 months, while for other breast cancer types it is about 5 years [30] and has shown limited improvement over time, despite the availability of numerous drugs [31]. Conversely, those diagnosed with metastatic hormone receptor-positive (HR-positive) and HER2-negative BC tend to experience a more extended median overall survival, often exceeding 2 years and occasionally extending even further. For patients with metastatic HER2-positive BC, the development of HER2-targeted therapies has yielded enhanced survival rates, pushing the median overall survival beyond the 2-year mark. Meanwhile, patients confronting metastatic BC characterised by both HR positivity and HER2 positivity face varied outcomes contingent upon the specific treatments administered and the distinct characteristics of the cancer, along with a consideration of the 3-year overall survival [32].

### 2.4. Need for New Treatment Approaches

Chemotherapy is a fundamental systemic treatment option for BC, encompassing endocrine therapy for HR-positive disease, chemotherapy, and more recently, immunotherapy for early TNBC disease [1]. Despite the current array of treatment options covering the diverse spectrum of BC, several questions remain regarding tailoring, optimising, and escalating or de-escalating treatment strategies to effectively manage the disease [31]. Traditionally, curative breast surgery and axillary sampling have served as primary therapeutic interventions for breast tumours. Histopathological examination and IHC evaluation of HR, HER2, and proliferation markers are currently used to identify BC subtypes [33]. Metastatic TNBC (mTNBC) is an aggressive subtype characterised by a one-year median survival rate and worse disease-specific survival compared to HR-positive subtypes. In contrast, patients with early-stage TNBC (I–III) often receive NAC [34]. Taxanes and anthracycline-based chemotherapy regimens form the cornerstone of TNBC treatment [34]. While anthracycline/taxane-based regimens are generally considered the standard of care, determining the optimal chemotherapy strategy for TNBC remains challenging due to insufficient evidence [35]. Taxanes disrupt microtubule dynamics and mitosis, impeding tumour growth, while anthracyclines induce DNA double-strand breaks [16]. The potential long-term cardiotoxicity associated with anthracycline-containing regimens cannot be ignored. Consequently, several trials [35,36] have investigated substitute chemotherapy medications as replacements for anthracyclines once taxanes were established as a viable treatment choice. However, there have been conflicting findings in different studies regarding the efficacy of regimens centred around anthracyclines and taxanes [16].

#### 2.4.1. Neoadjuvant Chemotherapy versus Adjuvant Chemotherapy for TNBC Patients

Despite the poor prognosis associated with TNBCs, some of these tumours exhibit high sensitivity to chemotherapy, making systemic NAC the standard treatment option for early-stage TNBC. NAC is an integral part of multidisciplinary therapy for BC [37], aiming to downsize the tumour before surgery and increase rates of breast conservation surgery [1]. Additionally, NAC allows for the evaluation of pCR, which provides important prognostic information and guides adjuvant treatment decisions. Patients who achieve a pCR, defined as the absence of invasive cancer in both the breast and regional lymph nodes within the surgically removed specimen, have better disease-free (DF) and OS outcomes [28,31,33,37]. Several neoadjuvant studies in TNBC have demonstrated that pCR is predictive of improved long-term outcomes and serves as a guide for further treatment decisions [31,38]. The International Working Group for Collaborative Trials in Neoadjuvant BC (CTNeoBC) conducted a pooled analysis of 12 neoadjuvant clinical trials, which revealed improved event-free survival (EFS) and OS associated with pCR, particularly in TNBC patients [39]. As a result, pCR has become an accepted surrogate endpoint in neoadjuvant clinical trials [28]. Moreover, the St. Gallen consensus conference has recently established NAC as the preferred treatment approach for TNBC patients, regardless of tumour diameter or axillary nodal involvement [40]. This decision was based on the significantly better EFS and OS observed in TNBC patients who achieved pCR compared to those with residual invasive disease following NAC [41].

Despite the clear association between pCR and prognosis, accurately predicting pCR before surgery remains challenging due to the complexity of multiple factors, including tumour biology, NAC protocols, and breast imaging results. While the overall response to chemotherapy in TNBC is modest, a subset of patients demonstrates excellent response rates compared to HR-positive and HER2-positive BC. However, these TNBC patients also experience higher rates of relapse at earlier intervals [29,42]. Therefore, the development of a biomarker to predict NAC response is crucial to guide treatment decisions and increase the likelihood of achieving pCR [37]. In efforts to increase pCR rates, studies have explored strategies for modifying treatment based on early response to NAC. A small study involving patients with large or locally advanced BC investigated this approach. Patients received four cycles of anthracycline-based polychemotherapy, and their response was assessed. Responding patients who achieved a partial response (PR) or pCR were randomised to continue the same treatment or switch to docetaxel monotherapy. Patients who did not achieve objective tumour regression received four cycles of docetaxel. The study yielded two key findings: switching to a non-cross-resistant regimen doubled the pCR rate in responding patients from 15% to 31%, while the pCR rate in non-responding patients receiving docetaxel remained low at 2% [31].

NAC has historically been used to convert patients with unresectable, locally advanced BC into surgical candidates, and more recently, its role has expanded to facilitate breast conserving therapy in patients with large, operable tumours who would otherwise require mastectomy [41]. A meta-analysis of 10 studies involving 4756 women revealed that NAC increased the rate of breast conserving therapy from 49% to 65% compared to adjuvant chemotherapy [43]. However, there is still a lack of prospective clinical trial data assessing the effectiveness of NAC in converting patients who are initially ineligible for breast conserving therapy into candidates, as well as limited studies investigating the relationship between preoperative evaluations for breast conserving therapy eligibility, genetic testing, and pCR [41].

TNBC patients exhibit higher response rates to chemotherapy due to their higher proliferation and chemosensitivity. Standard anthracycline and taxane-based regimens yield pCR rates ranging from 30% to 40% in TNBC [31]. Various novel agents and combination chemotherapy regimens are being studied to improve patient outcomes and maximise pCR rates [33], such as KEYNOTE-522 [44], NeoSTAR [45], and GeparNuevo [46]. Additionally, immune checkpoint inhibitors (ICI), which have revolutionised the treatment of several challenging cancers, have been investigated as part of neoadjuvant regimens for TNBC. The addition of the anti-programmed cell death receptor 1 (PD-1) monoclonal antibody pembrolizumab to a sequential weekly chemotherapy regimen involving carboplatin, paclitaxel, and anthracyclines has shown statistically improved pCR rates and a trend towards improved event-free survival. In conclusion, the expected pCR rate in TNBC using currently available NAC approaches is approximately 50%. With the addition of pembrolizumab, this rate may reach 60–70% in appropriately selected patients [47]. However, despite the high pCR rates observed, TNBC patients without pCR have a significantly poorer prognosis, with a high risk of early metastatic progression. In contrast, patients who achieve pCR exhibit relatively good outcomes [31]. On the other hand, adjuvant chemotherapy for TNBC follows the general principles applied to other phenotypes of BC. The current recommendations for early-stage TNBC do not differ from those for hormone-positive BC. Adjuvant chemotherapy is typically recommended for the early stages (I–III) of BC [20,29]. The achievement of pCR after NAC has been established as a significant prognostic marker and is used to guide adjuvant therapy decisions. Given the poor outcomes observed in patients with residual disease (RD) after NAC, various systemic therapeutic approaches in the adjuvant setting have been investigated. One notable trial is the Capecitabine for Residual Cancer as Adjuvant Therapy (CREATE-X) trial [48]. This study randomised HER2-negative BC patients who had RD after NAC to receive either capecitabine (1250 mg/m^2^ twice daily for up to 8 cycles) or placebo. The results showed that adjuvant capecitabine significantly improved 5-year DFS and OS rates compared to placebo (74.1% vs. 67.6% and 89.2% vs. 83.6%, respectively). Additionally, the use of platinum agents as an adjuvant treatment has shown promise in TNBC. These agents have been found to increase the percentage of patients achieving pCR and improve outcomes in TNBC patients. The addition of platinum agents resulted in a higher DFS rate (69.8% vs. 56.1%) and improved OS (78.8% vs. 70.3%) compared to non-platinum-based regimens [16]. The potential benefit of adding capecitabine to standard adjuvant chemotherapy in improving the prognosis of early-stage TNBC has been suggested by several studies [49,50]. However, it should be noted that capecitabine has not yet been incorporated into clinical guidelines for TNBC treatment. Ongoing trials are being conducted to further investigate adjuvant chemotherapy approaches and explore the effectiveness of novel agents in the management of TNBC with RD [16,28]. These trials aim to provide additional evidence and insights for optimising treatment strategies and improving outcomes in this patient population. Table 3 highlighted a summary of clinical trials in TNBC, design, and outcomes.

#### 2.4.2. Immunotherapy

Immunotherapy has emerged as a promising approach in cancer therapy, particularly in the context of the tumour microenvironment’s impact on tumour progression and metastasis. TNBC, based on multiple subtype classifications, has been shown to have an immune-related subtype in approximately 20% to 30% of patients, indicating the potential effectiveness of immunotherapy as a treatment option [16].

Tumour-infiltrating lymphocytes (TILs) are immune cells, including cytotoxic CD8+ lymphocytes and CD4+ T helper cells, that infiltrate tumour tissues. The presence of TILs has been considered a prognostic biomarker in TNBC due to their high prevalence in this subtype. In patients receiving NAC, increased TIL concentration has been associated with better survival in HER2-positive BC and TNBC but worse survival in ER positive/HER2-negative BC. Notably, two important analyses have highlighted the significance of stromal TILs (sTILs) in early-stage TNBC, suggesting that sTILs could identify a subgroup of TNBC patients with excellent prognosis who may not require adjuvant chemotherapy. Furthermore, the presence of TILs in RD following NAC has been associated with better outcomes, and their presence during neoadjuvant anthracycline/taxane chemotherapy is linked to a higher rate of pCR. These findings emphasise the potential of TILs as prognostic and predictive markers in TNBC [16,51]. While immunotherapy has shown benefits for TNBC patients and has gained significant attention in recent years, strong evidence supporting its use in the adjuvant setting is currently lacking. Further validation and research are necessary before immunotherapy can be established as a standard adjuvant treatment for TNBC patients.

#### 2.4.3. Local Therapy: Surgery and Radiation

In addition to chemotherapy, adjuvant radiotherapy (RT) can also play a crucial role in improving the prognosis of TNBC patients. Local management strategies, including surgery and radiation, are implemented similarly across all BC subtypes, without specific recommendations unique to TNBC [29]. Both breast-conserving surgery (BCS) [11] and mastectomy are viable options for the surgical treatment of early-stage TNBC patients [52].

BCS, followed by adjuvant radiation treatment, is the standard approach for early-stage BC. Numerous randomised trials over the past 50 years have demonstrated that radiation treatment significantly reduces locoregional recurrence after BCS, resulting in high rates of breast preservation and excellent survival outcomes [53]. However, BCS may not always be feasible, and there has been a reported increase in mastectomy rates despite strong evidence demonstrating non-inferior survival outcomes with BCS compared to mastectomy [54]. In the United States, the proportion of mastectomy procedures has been rising, particularly among TNBC patients, with over 50% of women with operable TNBC opting for mastectomy instead of breast-conserving therapy [55]. Several factors contribute to the high mastectomy rates, including the higher prevalence of germline mutations, tumour location, the relationship between tumour size and intact breast size, family history of BC (common factors in TNBC), and the availability of various breast reconstruction options [29,56]. On the other hand, a study conducted in China compared the prognoses of TNBC patients with early-stage disease who underwent BCS plus radiotherapy (BCS + RT), mastectomy alone, or mastectomy plus RT to evaluate whether the scope of surgery could be reduced for these patients [52]. The study found that BCS + RT yielded better outcomes and that BCS is more acceptable to TNBC patients due to its impact on post-surgical quality of life and advancements in medical technology [52]. The lack of clear conclusions regarding the choice between BCS and mastectomy in these studies may be due to geographic differences. While BCS is the preferred option for the majority of early-stage BC, a significant percentage of women still choose mastectomy as their initial surgical approach. However, for certain subgroups of BC patients, the risk of local recurrence remains even after mastectomy. Therefore, studies have been conducted to evaluate whether postmastectomy radiation therapy (PMRT) should be considered for specific subgroups of women with early BC [56,57]. PMRT was initially recommended for women with locally advanced tumours in the breast and is not routinely indicated for early breast tumours treated with mastectomy [56]. However, determining which patient would benefit from PMRT continues to pose questions. Therefore, conducting well-controlled studies is crucial to ensuring optimal treatment while minimising over- and infra-treatment scenarios for this group of patients.

## 3. Role of Biomarkers in TNBC

Biomarkers are defined by the National Cancer Institute (NCI) as biological molecules present in blood, other bodily fluids, or tissues that serve as an indicator of either a normal or a specific condition or disease [58]. In clinical oncology practice, biomarkers play a crucial role as they provide measurable biological variables that can be used to predict survival or response to treatment interventions. They are also essential in early diagnosis, treatment monitoring, and personalised drug information in translational research discussions. However, the integration of biomarkers into clinical practice requires rigorous laboratory and clinical validations through well-designed clinical trials [59]. In addition to TNBC’s aggressive nature, it shows limited sensitivity to endocrine agents and a lack of targeted therapy options, leading to significantly shorter DFS and OS. Therefore, alternative therapeutic strategies are urgently needed [60,61]. The incorporation of chemotherapy in the treatment of high-risk early-stage TNBC has shown significant reductions in disease recurrence rates. However, this progress comes with potential toxicities, including cardiac risks, life-threatening infections, and long-term peripheral neuropathy, which can impact patients’ function and quality of life. Therefore, it is crucial to make informed decisions considering the risks and benefits of adjuvant therapies [60]. Traditionally, prognostic and adjuvant therapy decisions for early-stage TNBC have relied on clinicopathologic factors such as tumour burden, grade, size, and proliferative index, as well as patient factors such as age and additional health issues [60]. However, TNBC patients exhibit a wide range of clinical outcomes, including varying rates of pCR after NAC in early-stage disease and diverse responses to therapy and subsequent survival in the metastatic setting [62]. While genomic assays have provided additional tools to guide treatment recommendations in specific disease phenotypes, the current landscape of clinical trials and immunotherapy lacks a reliable and reproducible biomarker to facilitate decision-making, highlighting the need for further research in this area [60].

### Importance of Predictive Biomarkers

**Figure 2:**
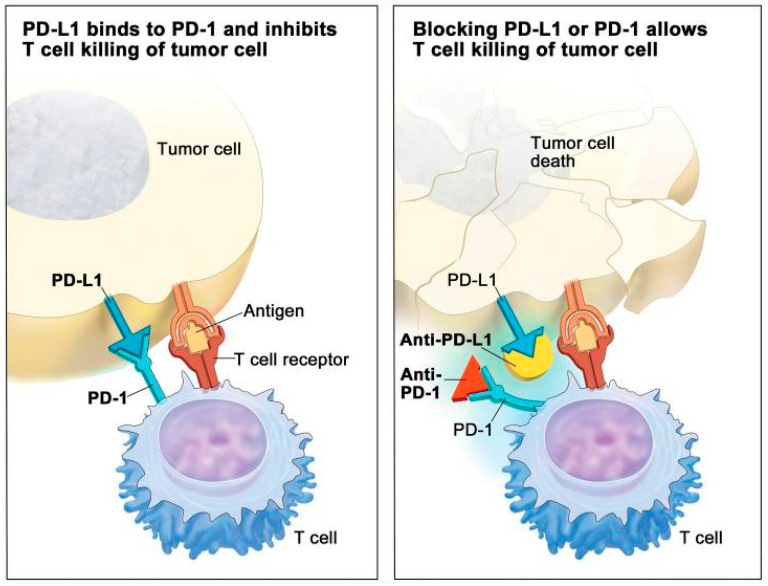
PD-1 and PD-L1 inhibitors’ mechanisms of action. Tumour cells produce PD-L1 to bind with PD-1 on T cells, preventing T cells from killing tumour cells. T cells are able to kill tumour cells by blocking PD-L1’s ability to bind to PD-1 with a PD-1 or PD-L1 inhibitor. Figure adopted from Eno, MS, PA-C, 2017 [69].

Predictive biomarkers guide treatment decisions by identifying which patients are likely to respond to specific chemotherapeutic agents, such as those predicting sensitivity to platinum-based therapies. Predictive biomarkers enable personalised treatment plans, improving outcomes by selecting therapies based on the tumour’s molecular and genetic profile. They also have prognostic value, influencing long-term management strategies, and are essential for designing biomarker-driven clinical trials and accelerating the development of new therapies. Key predictive biomarkers include BRCA1/2 mutations, which are present in about 15–20% of TNBC cases and indicate [63]. Studies have shown that gBRCA TNBC patients exhibit improved OS, which may be attributed to their increased sensitivity to chemotherapy due to enhanced immune activation [62]. Additionally, the expression of PD-L1 serves as a biomarker for the efficacy of immune checkpoint inhibitors, providing new treatment avenues with drugs such as pembrolizumab. Blocking the PD-1/PD-L1 interaction with anti-PD-L1 therapeutic agents prevents the deactivation of CD8+ T-cells (Figure 2) and enhances the immune response against cancer cells, leading to tumour regression [64]. While PD-L1 expression in BC is associated with aggressive behaviour and poor survival, its prognostic role in TNBC remains unknown [65]. Recent studies have shed light on the potential benefits of targeting PD-L1 in TNBC. For instance, Li et al. [66] examined PD-L1 expression in a cohort of 136 TNBC cases/patients and found that 51% of them showed positivity in tumour/stromal PD-L1, which according to multivariate analysis was associated with improved DFS [67]. Similar findings were reported by Botti et al. (2017) [68]. However, further confirmation through larger cohort studies is warranted.

Additionally, the presence of TILs and the immune response are believed to be critical in therapeutic approaches [65]. High levels of TILs in TNBCs have shown a significant prognostic impact and a reduction in distant metastasis [70]. Several studies have consistently found that a high degree of lymphocytic infiltration is associated with better outcomes in early-stage TNBC patients [71,72]. Additionally, TILs have been suggested as a potential source of other biomarkers, such as the recently reported PD-L1 [59,73,74]. Therefore, TILs should be regarded as a key biomarker for immunotherapy and chemotherapy in early TNBC. In the NAC of early TNBC, a pooled analysis of 3771 patients treated with NAC combination chemotherapy from six randomised trials conducted by the German BC Group revealed that a 10% increase in TILs was associated with longer DFS and OS. The pCR rate was 31% in patients with low TILs and 50% in patients with high TILs [74,75]. These findings provide a strong rationale for combining chemotherapy and immunotherapy in the neoadjuvant setting of TNBC to increase the pCR rate and improve survival outcomes. TILs in BC specimens at diagnosis or following NAC or adjuvant chemotherapy have been shown to be important prognostic and predictive factors in TNBC. Moreover, TIL levels in RD after treatment can provide additional prognostic information [76]. Incorporating RD TILs into clinical trials may help refine neoadjuvant endpoints and potentially identify patients who could benefit from additional adjuvant chemotherapy or immunotherapeutic interventions. Despite challenges such as TNBC’s heterogeneity and the need for comprehensive biomarker panels, the integration of predictive biomarker testing into routine clinical practice promises to enhance therapeutic precision and effectiveness for TNBC patients.

## 4. Mass Spectrometry-Based Proteomics in TNBC

MS platforms are powerful analytical tools that have revolutionised the field of proteomics. They allow for the identification, quantification, and characterisation of proteins within complex biological samples. MS enables the measurement of the mass-to-charge ratio (*m*/*z*) of ions generated from peptides/proteins, providing information about their molecular weight, sequence, post-translational modification (PTMs), and interactions [77]. The importance of resolution and mass accuracy cannot be overstated; resolution refers to the instrument’s capacity to distinguish ions with similar *m*/*z* values, with higher resolution enhancing ion separation and mass determination precision. In the field of tandem mass spectrometry (MS/MS), specific ions identified in the initial mass analysis undergo further fragmentation, yielding daughter ions that reveal the molecular structure and sequence of biomolecules such as proteins and peptides. Moreover, mass accuracy correlates to the proximity of a measured ion’s mass to its actual mass, an aspect critical for accurate identification and quantification.

MS-based proteomics facilitates an extensive examination of the proteome, enabling the identification and quantification of thousands of proteins within TNBC tissues, blood samples, and cell lines. This technique has been crucial in discovering protein biomarkers and pathways such as the PI3K/AKT/mTOR and EGFR that are dysregulated in TNBC. By analysing the protein profiles of TNBC, researchers can identify proteins that are distinctly expressed or modified in TNBC compared to other BC subtypes, which can lead to the identification of potential therapeutic or diagnostic targets. Additionally, this approach helps in understanding tumour heterogeneity, which is linked to the varying clinical outcomes observed in TNBC. Table 4 provides a summary view of the protein biomarkers identified through MS-based proteomics in TNBC.

### 4.1. Technology Overview

**Figure 3:**
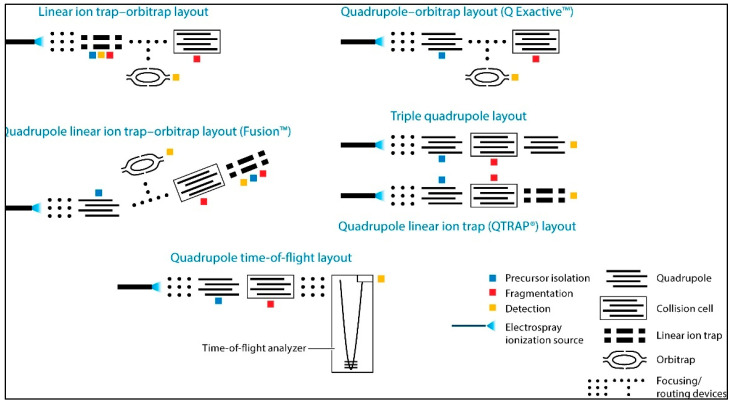
A schematic overview of different hybrid MS designs used in modern proteomics research. Each coloured square indicates the occurrence of three important experimental steps: precursor isolation (highlighted in blue), fragmentation (highlighted in red), and detection (highlighted in yellow). Figure adopted from Gillet, Leitner, and Aebersold, 2016 [90].

All MS are composed of three linked units: an ionisation source, an analyser, and a detector, which are linked to a computer system to provide a measurement of abundance for the ions/spectra detected [87]. Ionisation source: The emergence of MS-based proteomics has been greatly facilitated by the development of ionisation techniques, with notable examples being electro-spray ionisation (ESI) and matrix-assisted laser desorption ionisation (MALDI). Ionisation techniques in MS refer to ionisation approaches that result in minimal fragmentation of analyte molecules during the ionisation process. ESI, in particular, is widely used in combination with reversed phase nano/LC technologies or capillary electrophoresis for efficient analysis of complex protein samples [58]. While MALDI works by combining the analyte of interest with the appropriate matrix material. The matrix absorbs laser energy and contributes to ionisation [88]. Mass analysers: Mass analysers are integral components of MS instruments that separate ions based on their *m*/*z* ratio. These analysers are critical in determining ion mass and obtaining precise mass measurements in a variety of analytical applications. Common types include ion trap: which consist of a three-dimensional electrostatic field that traps ions. Ions are trapped and detected based on their *m*/*z* ratio and trap stability, offering high sensitivity and multiple-stage fragmentation capability [89]. Time of flight (TOF): measuring the time it takes ions to travel a fixed distance from the ion source to the detector. This travel time corresponds to the ions’ *m*/*z* ratio, allowing their mass to be calculated, providing accurate mass measurements, fast data acquisition, and low fragmentation. Quadrupole: consists of four parallel rods that create a radiofrequency (RF) and direct current (DC) electric field. These electric fields control the trajectory of ions, allowing specific ions to pass through the analyser while filtering out others. It is known for its selectivity, sensitivity, and rapid scanning speed. Orbitrap: operates on the Fourier Transform Ion Cyclotron Resonance (FT-ICR) principle with a unique electrode design for precise *m*/*z* ratio measurement [87]. Hybrid instruments, such as Q-TOF [87] and Q-Orbitrap [90], combine different analysers for comprehensive structural elucidation and molecule identification; see Figure 3 [90]. Detector: The detector is the final important component of the MS that converts ion signals into measurable electrical signals. It detects and measures the abundance of ions generated in the ion source and separated by the mass analyser. The primary function of the detector is to generate a signal that represents the ion current or ion abundance at various *m*/*z* ratios for subsequent data analysis [91].

### 4.2. Identification of Protein Biomarkers in TNBC

Proteins can be analysed using MS through a method known as bottom-up proteomics, which is, in fact, the most commonly employed approach in the field. In bottom-up proteomics, proteins undergo enzymatic digestion to break down into peptides. Subsequently, these peptides are separated and subjected to analysis using MS for identification and quantification [92]. Presently, bottom-up methodologies employing data-dependent acquisition (DDA) workflows serve as the foundational technologies in proteomics. These straightforward approaches, also referred to as shotgun proteomics, yield extensive lists of protein identifications and have been instrumental in deciphering complex, comprehensive proteomes, including the initial versions of the human proteome [93]. Concurrently, data-independent acquisition (DIA) is evolving as a prominent method for the future, aiming to merge a high volume of protein identifications with the consistent and accurate quantification of protein levels [92,93]. Discovery proteomic: the practice of discovery proteomic analysis, commonly referred to as shotgun proteomics, has revolutionised the field by providing an effective method for achieving unbiased and comprehensive coverage of the proteome. This powerful approach enables the identification and quantification of a large number of proteins within a sample (potential biomarkers), allowing researchers to investigate complex protein mixtures and gain insights into cellular processes on a global scale [94].

### 4.3. Current Gaps in Biomarker Discovery

**Figure 4:**
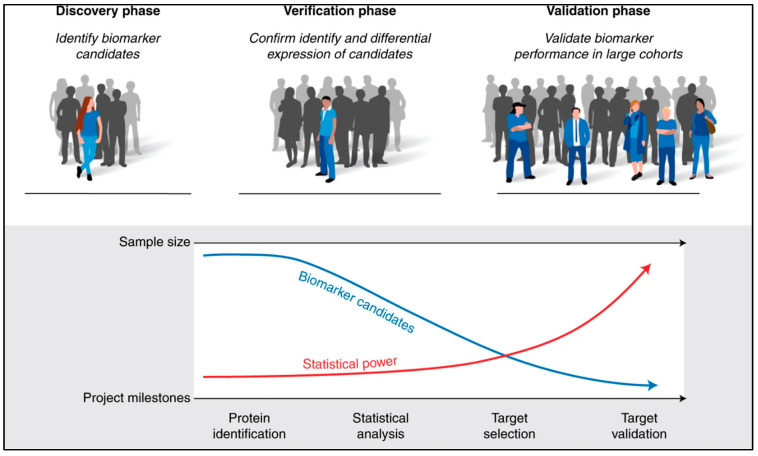
Phases of biomarker development studies. Biomarker development is typically divided into three stages: discovery, verification, and validation. During the discovery phase, a small number of samples are submitted for in-depth proteomics analysis, which involves measuring thousands of proteins to identify biomarker candidates. In subsequent phases, larger cohorts of samples are frequently analysed, increasing statistical power. Biomarker candidates are also narrowed down at each developmental stage based on their ability to accurately predict the disease or condition. As a biomarker, a combination of proteins rather than individual proteins is tested in some cases. Biomarker candidates are subjected to additional proteomics analysis in the verification phase to confirm their identities and expression in the same or similar samples as in the discovery phase. During the validation phase, a few of the most promising candidates are tested to determine their suitability for clinical use. Figure taken from Nakayasu et al., 2021 [95].

The process of biomarker development is commonly divided into three stages: discovery, verification, and validation (Figure 4) [95]. To achieve comprehensive proteome coverage during the discovery phase, in-depth proteomics analysis using LC-MS/MS is conducted on a small number of samples. Strategies such as abundant protein depletion and peptide prefractionation are employed to enhance the detection of low abundant proteins, depending on the sample complexity. However, due to the high cost, logistical challenges, and low throughput of discovery proteomics, this phase is typically carried out with a limited number of samples. Furthermore, ensuring the reliability of initial identifications made during the discovery phase requires confirmation in the same or similar samples used in the discovery phase, as the identification relies on matching experimental MS/MS spectra to computationally predicted MS/MS spectra [95]. Unbiased discovery in proteomics has led to the identification of numerous potential biomarkers and the characterisation of protein expression patterns in various diseases and biological processes. However, despite significant progress in the discovery of proteomics research, the number of protein biomarkers used in clinical practice remains relatively small. There are several scientific and procedural gaps between the discovery proteomics research and clinical implementation, which contribute to the limited validity and clinical applications of biomarkers. Additionally, the complexity and low throughput of proteomics approaches pose additional barriers to translating biomarker assays into clinical applications. Further verification and validation studies are required to confirm the reproducibility and clinical significance of the discovered candidates. In this context, targeted proteomics has emerged as a powerful tool to bridge the gap between biomarker discovery and clinical validation [96].

## 5. Clinical Implications

### 5.1. Advances in Clinical Practices through Biomarker Research and Proteomics

Clinical practices are being enhanced through research focused on early detection methods, risk group classification, and treatment efficacy. A system biology approach is employed to discover biomarkers and characterise tumours at the molecular level. Routine biomarkers, such as specific proteins, genes, or hormones, measurement, and improved treatment options, have contributed to a gradual decrease in cancer mortality rates, with an estimated annual decline of 2.9 million cases and a 29% decrease over the past three decades [97]. Another area of clinical research aims to identify molecular differences between cancer cases and healthy controls, as well as different stages of cancer progression. Genomics and transcriptomics have uncovered numerous cancer-causing genes. While these omics datasets enable comparisons between different clinical cancer groups, they do not always directly translate to our understanding of disease biology. In contrast, proteins, which actively carry out biological processes, serve as ideal predictors of disease progression [98]. Proteins are also the primary targets of cancer therapeutics, including antibody-based therapies. Clinical proteomics, which involves the large-scale study of proteins (expression, function, structure) and their application to improve patient care, is a growing field in molecular clinical research [58]. Processes such as alternative splicing, single nucleotide polymorphisms (SNPs) leading to different proteoforms, transcript degradation, protein-protein interactions, degradation rates, and PTMs are covalent chemical modifications that occur after a protein is synthesised during translation, typically in the cytoplasm or on ribosomes, and all influence protein expression and protein function [99]. Accurate protein detection techniques are essential for routine clinical analysis [58].

In clinical proteomics, there is currently a strong reliance on antibody-based techniques. Protein biomarkers are often quantified in various biofluids and tissues using Western blotting, enzyme-linked immunosorbent assay (ELISA), and immune histochemistry (IHC). For example, the protein markers HER2, ER, and PR are used to classify BC subtypes, impacting treatment decisions. IHC can also identify tissue physiologies associated with poor prognosis or treatment response [58,66]. Fluorescence-activated cell sorting (FACS) utilises antibodies to detect heterogeneity within a cell population by targeting a small panel of protein markers [58]. While antibodies offer high specificity, their application is limited by factors such as development cost, availability, quality, and multiplexing capability. The need for higher throughput techniques that capture a broader range of the cancer proteomic landscape has led to the emergence of mass spectrometry (MS)-based techniques in oncology clinics. Technological advancements in sample preparation, peptide separation, MS detection, and data analysis have been crucial in accurately quantifying proteins from complex clinical samples [58,66]. Recent advancements in MS-based proteomics have generated a vast amount of proteomic data, enabling the identification of protein signatures or biomarkers that can improve cancer diagnosis, prognosis, and treatment. [66]. Recently, proximity extension assay (PEA) technology has gained widespread adoption in both research and clinical settings for protein biomarker discovery [100]. This technology offers highly sensitive, targeted immunoassay, multiplexing capabilities, and compatibility with various sample types. PEA is an emerging technology introduced by Olink proteomics (in Uppsala, Sweden) [101], and it combines quantitative real-time PCR (qPCR) with multiplex immunoassays. PEA employs a matched pair of antibodies labelled with unique DNA oligonucleotides for the dual recognition of a targeted biomarker [100]. The quantification of biomarker-specific DNA “barcodes” is quantified by microfluidic qPCR [101].

### 5.2. The Advantages and Disadvantages of MS-Based Proteomics in TNBC

MS-based proteomics provides valuable insights into the proteome of TNBC and holds promise for advancing the understanding and treatment of the disease. However, challenges related to data complexity, cost, and sample preparation are also faced. The advantages and limitations of MS-based proteomics for clinical applications are summarised following.

#### 5.2.1. Advantages of MS-Based Proteomics in Clinical Applications

- Comprehensive protein profiling: MS-proteomics allows for the identification and quantification of a wide range of proteins in TNBC tissues. This extensive profiling can uncover novel biomarkers for cancer diagnosis, prediction, and prognosis, which are increasingly sought to enable early cancer detection and to tailor treatment decisions [102].
- Post-translational modifications: Protein PTMs significantly impact protein functions and are vital to nearly all cellular processes. PTMs and their interactions are closely associated with key signalling events that drive cancer development, progression, and metastasis. They play crucial roles in cancer hallmark functions, cancer metabolism, and the regulation of the tumour microenvironment [103]. As a result, by studying PTMs through MS-proteomics, researchers can uncover specific modifications that are associated with TNBC, providing insights into how these changes drive the disease. This knowledge can lead to the identification of novel therapeutic targets and the development of drugs that specifically inhibit or modify these PTM-related pathways. Additionally, PTMs can serve as biomarkers for disease progression and treatment response, further enhancing the ability to tailor treatments to individual patients [66].
- High sensitivity and specificity: MS-proteomics provides high sensitivity and specificity, making it a powerful tool for detecting low-abundance proteins. This capability is especially critical in the context of TNBC, where key proteins involved in signalling pathways, tumour suppression, and drug resistance may be present in very low quantities. The ability to identify these proteins can lead to the discovery of novel biomarkers that could be crucial for early detection and personalised treatment strategies [104]. Moreover, MS-proteomics helps in distinguishing between closely related protein isoforms, which is important for understanding the variations in protein structure and function that can influence TNBC progression and treatment response. Protein isoforms can arise from alternative splicing, PTMs, or genetic mutations, and each isoform may play a distinct role in the disease. By accurately identifying and quantifying these isoforms, researchers can gain deeper insights into the molecular heterogeneity of TNBC, leading to more targeted and effective therapeutic approaches [105].
- Integration with other omics data: MS-proteomics data can be integrated with genomics and transcriptomics data to offer a more comprehensive understanding of TNBC biology. This integrative approach helps to elucidate the complex interactions between proteins, genes, and RNA transcripts, providing a deeper understanding of the disease’s molecular mechanisms. For instance, the Human Protein Atlas (HPA) serves as a valuable resource, offering detailed information on the localisation and temporal expression of human proteins across various tissues and cancers. It also provides insights into the availability and quality of antibodies, which can be cross-referenced with genomic and transcriptomic data to link protein behaviour with gene expression patterns [106]. Similarly, the Clinical Proteomic Tumour Analysis Consortium (CPTAC) of the National Cancer Institute (NCI) has made significant contributions by publishing comprehensive multi-omics studies, including MRM (Multiple Reaction Monitoring) assay databases for several cancer types, such as breast [23] and ovarian cancers [107]. The data available through the CPTAC Data Portal include protein sequence databases derived directly from the exome sequences of respective cancer samples, facilitating the integration of proteomic data with genetic information [108].
- Potential for personalised medicine: The advent of personalised medicine offers significant promise for those affected by this challenging disease, as distinct, potentially druggable molecular targets with unique alterations have been identified. Developing treatment strategies for a broad range of TNBC patients requires a thorough understanding of the disease’s underlying mechanisms. Achieving this understanding involves examining and integrating data on TNBC subtypes, focussing on their epigenetic, transcriptomic, proteomic, and phospho-proteomic profiles. For instance, a study has analysed the BRCA1-wild-type MDA-MB-231 TNBC cell line, the BRCA15382insC HCC1937 TNBC cell line, and the MCF10A cell line as a normal breast epithelial control. This multi-omics approach underscores the diversity among different TNBC subtypes and enhances the understanding of the molecular pathways that drive this complex form of BC [102].
- Multiplexing capabilities: MS-based assays can analyse multiple biomarkers simultaneously [109], which is highly efficient and cost-effective compared to other techniques such as enzyme-linked immunosorbent assay (ELISA), which typically measures one biomarker at a time.
- Application to various sample types: Advancements in mass spectrometry technologies, along with enhanced sample preparation techniques, have significantly improved our understanding of the biological complexity across a diverse range of sample types. This includes various organelles, membranes, biofluids (such as blood, cerebrospinal fluid, saliva, and urine), tissues, organs, and microbial communities [110].

#### 5.2.2. Disadvantages of MS-Based Proteomics in Clinical Applications

- Complexity and cost: MS-based proteomics relies on advanced and costly equipment, including high-resolution mass spectrometers. These devices require frequent maintenance and calibration to maintain their precision and dependability. Operating and maintaining such instruments demands specialised training and expertise. Additionally, methods for absolute quantification, such as TMT and iTRAQ, used in TNBC biomarker studies, contribute to further expenses [111].
- Sample preparation challenges: Sample preparation plays a critical role in the proteomic characterisation of clinical samples, and it is essential to establish rigorous standard operating procedures to obtain relevant information about the complex biological processes underlying cancer progression. There is no universal protocol for proteomic sample preparation, as the chosen strategy should be optimised based on factors such as proteomic complexity, available sample quantity, and the study’s goals. Variations in sample handling, storage, and processing can affect the reproducibility and reliability of results [58].
- Data analysis complexity: MS generates vast quantities of data that necessitate the use of advanced analytical techniques and bioinformatics tools for meaningful interpretation. The initial step in data analysis involves the accurate identification and quantification of proteins, a process that relies on advanced algorithms and specialised software such as MaxQuant (version number v2.6.3.0) [112]. This includes the extraction of peptide sequences, matching them against protein databases, and quantifying their abundance. Furthermore, the reliability of results is contingent upon the ac-curacy of the analytical methods used. Limitations in data analysis can significantly impact the interpretation of results, potentially leading to misleading conclusions [113].
- Limited standardisation: Despite significant advancements in technology development, standardisation, and bioinformatics that have enhanced the reliable identification of molecular disease signatures, several major obstacles continue to hinder the effective translation of protein candidates into clinical biomarkers. As a result, only a limited number of biomarkers have received FDA approval in the past two decades [114].
- Validation and translation: The validation of protein biomarkers identified through MS requires extensive testing across different TNBC patient cohorts to ensure that the biomarkers are consistent, reliable, and reflective of the disease state. This process is complicated by the heterogeneity of TNBC, where the variability between tumours can lead to inconsistent biomarker performance [114]. Even when a protein biomarker is validated, translating it into a clinical setting involves overcoming several hurdles. Regulatory approval processes, such as those required by the US Food and Drug Administration (FDA), demand rigorous evidence of clinical utility and cost-effectiveness. Additionally, developing standardised and scalable assays for routine clinical use can be technically challenging and resource-intensive. As a result, despite the identification of promising biomarkers through MS-based proteomics, only a few have successfully made the transition to clinical practice in TNBC. Most FDA-approved tumour markers are blood-based and are used in conjunction with standard imaging techniques to differentiate between malignant and benign conditions. However, many existing cancer screening tests suffer from insufficient sensitivity and/or specificity. As a result, the search for protein biomarkers capable of enabling early cancer diagnosis remains an ongoing effort [114].

### 5.3. Need for Biomarkers in Predicting the Response to Neoadjuvant Chemotherapy

The necessity for biomarkers to predict the response to NAC in TNBC is of utmost importance for optimising patient care and improving outcomes. Protein biomarkers play a pivotal role in current medical research and clinical practice. These biomarkers are measurable indicators that reflect the physiological and pathological processes occurring within the body. The analysis of protein biomarkers within serum has facilitated notable progress in the fields of accurate diagnosis, screening, and early detection of cancer, prognosis, prediction of response to therapy, and a more profound comprehension of diverse illnesses, including cancer [115]. One of the key advantages of using serum protein biomarkers is the less invasive nature of obtaining a sample. Unlike obtaining tissue samples, serum can be collected through a simple blood draw, making it a convenient and widely accessible method for monitoring health and disease progression. In the domain of TNBC research, multiple studies have employed MS-based proteomics to explore novel diagnostic biomarkers [66,116]. Despite these efforts, credible and validated protein biomarkers suitable for clinical application have not yet emerged [24,117]. The ultimate achievement of successful biomarker development involves various factors, such as the identification of relevant biomarkers, optimisation of their selectivity and sensitivity, and clinical study design. Among these important factors, the accessibility of appropriate and sufficient clinical samples is essential for both the initial discovery phase and subsequent clinical evaluation and validation.

## 6. Conclusions

The urgency for innovative treatments for triple-negative breast cancer (TNBC) cannot be overstated. Current therapies often fall short due to the aggressive nature and molecular heterogeneity of TNBC. Thus, exploring new therapeutic approaches is not merely beneficial but essential. Cutting-edge techniques, such as targeted therapies, immunotherapies, and personalised medicine, hold promise for significantly improving patient outcomes. In parallel, mass spectrometry-based proteomics emerges as a pivotal tool in this quest. By providing an in-depth analysis of protein expressions and modifications, proteomics enhances our understanding of TNBC at a molecular level. This detailed proteomic profiling can identify novel biomarkers for early diagnosis, prognostic evaluation, and treatment personalisation for TNBC patients undergoing NAC, bridging critical gaps in current clinical practices. Consequently, integrating mass spectrometry-based proteomics with innovative treatment strategies offers a comprehensive approach to addressing the unmet clinical needs in TNBC, paving the way for more effective and personalised patient care.

## Figures and Tables

**Table 1:** A summary overview of key genes frequently altered in TNBC, along with their associated functions, frequency in TNBC, and clinical implications.

Gene	Function	Frequency in TNBC	Clinical Implications
TP53 mutation	Tumour suppressor gene	41% of tumours	The inactivation of tumour suppressor genes, resulting in the advancement of tumour growth.
PIK3CA mutation	Catalytic subunit of PI3K	30%	Activates the PI3K/AKT/mTOR pathway, promoting cell survival and growth.
MYC overexpression	Oncogene	20%	Stimulates cell division and supports tumour development.
PTEN inactivation	Tumour suppressor gene	16%	Activates the PI3K/AKT pathway due to the loss of regulatory inhibition.
BRCA1/BRCA2 Germline mutation	DNA repair, tumour suppressor genes	72%	Strong correlation with hereditary TNBC.
FGFR1 overexpression	Receptor tyrosine kinase	11%	Overexpression of FGFR1 contributes to tumour aggressiveness.

**Table 2:** Overview of molecular targets in TNBC. This table presents a summary of key molecular targets identified in Triple-Negative Breast Cancer (TNBC). It includes details on each target’s associated signalling pathway, available or potential treatments, clinical development status, and relevant biomarkers.

Molecular Target	Targeted Pathway/Process	Associated Therapy	Clinical Status	Remarks
PD-L1	Immune checkpoint inhibition	Pembrolizumab, Atezolizumab	FDA-approved for specific TNBC cases	Enhances immune response against tumours [22]
PARP1/2	DNA damage repair	Olaparib, Talazoparib	FDA-approved for BRCA-mutated TNBC	Exploits synthetic lethality in BRCA-deficient tumours [15]
EGFR	Growth factor signalling	Cetuximab (in trials)	Under investigation	Overexpressed in some TNBC subtypes [23]
Androgen Receptor (AR)	Hormone receptor signalling	Enzalutamide (in trials)	Under investigation	Targeted in AR-positive TNBC [24]
PI3K/AKT/mTOR	Cell growth and survival	Alpelisib (in trials)	Under investigation	Pathway frequently activated in TNBC [25]
CDK4/6	Cell cycle regulation	Palbociclib, Ribociclib (in trials)	Under investigation	Inhibition can block cell proliferation in TNBC [26]
BRCA1/2	DNA repair	Olaparib	FDA-approved for BRCA-mutated TNBC	Germline mutations can drive tumour development [27]

**Table 3:** Overview of Key Clinical Trials in TNBC: designs, populations, interventions, and outcomes. This table examines a range of therapeutic approaches, such as immunotherapy, chemotherapy, and targeted therapies. The trial design includes randomised controlled trials, single-arm studies, and phase II/III trials, with a focus on their clinical outcomes.

Trials	Design	Population	Intervention	Outcomes
KEYNOTE-522	Phase III, Randomised, Double-Blind, Placebo-Controlled	Early-stage, high-risk TNBC patients	Pembrolizumab + NAC vs. Placebo	Improved pCR and EFS, supporting pembrolizumab with NAC for high-risk TNBC.
NeoSTAR	Phase II, Single-Arm	Stage II/III TNBC patients	Neoadjuvant Nivolumab + Chemotherapy	Trial is ongoing. Promising pCR rates and immune activation, suggesting neoadjuvant nivolumab’s potential in TNBC, though further studies needed to confirm efficacy
GeparNuevo	Phase II, Randomised, Double-Blind, Placebo-Controlled	Early-stage TNBC patients	Neoadjuvant Durvalumab + Chemotherapy vs. Placebo	Durvalumab improved pCR rates, particularly when administered before chemotherapy, highlighting potential timing considerations for ICI in TNBC treatment.
CREATE-X	Phase III, Randomised, Open-Label	HER2-negative breast cancer patients with residual disease	Adjuvant Capecitabine vs. Observation	Significantly improved DFS and OS in patients with residual disease after NAC, demonstrating the benefit of adjuvant capecitabine, especially in the TNBC subpopulation.
ADAPT-TN	Phase II, Randomised	Early-stage TNBC	Neoadjuvant Pembrolizumab + Nab-Paclitaxel + Epirubicin vs. Chemotherapy Alone	Trial is ongoing. Evaluates the impact of adding pembrolizumab to NAC on pCR rates, with positive results suggesting enhanced efficacy in TNBC.
SASCIA	Phase III, Randomised, Open-Label	Patients with early-stage TNBC are at high risk of recurrence	Sacituzumab Govitecan vs. Treatment of Physician’s Choice	Trial is still ongoing. Aims to assess the efficacy of Sacituzumab Govitecan in reducing recurrence rates and improving survival in high-risk TNBC patients following standard neoadjuvant therapy.

**Table 4:** An overview of protein biomarkers identified in TNBC via MS-based proteomics. It includes details on the biomarker or pathway, its role in TNBC, clinical relevance, and references from MS-based studies.

Protein Biomarker/Pathway	Role in TNBC	Clinical Relevance	MS-Based Studies: References
EGFR	Growth factor signalling	Potential target for targeted therapy	Studies have confirmed that EGFR expression is elevated in TNBC, suggesting its potential as a therapeutic target [23,78].
PARP1/2	DNA damage repair	Targeted by PARP inhibitor	Studies have validated the critical role of PARP1/2 in BRCA-mutated TNBC, thereby guiding treatment strategies [79,80].
CXCL8 (IL-8)	Chemokine signalling	Linked to poor prognosis and metastasis	Studies have linked elevated CXCL8 levels to poorer clinical outcomes in TNBC patients [81,82].
PD-L1	Immune checkpoint regulation	Targeted by immune checkpoint inhibitors	Studies have revealed the upregulation of PD-L1, supporting the use of immune checkpoint blockade in TNBC [83,84].
MUC1	Cell surface glycoprotein	Potential target for immunotherapy	Identified as an overexpressed marker in TNBC, it is considered a potential target for novel therapies [66,85].
S100A7	Calcium-binding protein	Associated with invasion and metastasis	Identified as a key contributor to TNBC progression, particularly in its more invasive forms [86].
